# A novel variant in the *PDE4D* gene is the cause of Acrodysostosis type 2 in a Lithuanian patient: a case report

**DOI:** 10.1186/s12902-021-00741-6

**Published:** 2021-04-15

**Authors:** Gunda Petraitytė, Kamilė Šiaurytė, Violeta Mikštienė, Loreta Cimbalistienė, Dovilė Kriaučiūnienė, Aušra Matulevičienė, Algirdas Utkus, Eglė Preikšaitienė

**Affiliations:** 1 Department of Human and Medical Genetics, Institute of Biomedical Sciences, Faculty of Medicine, Vilnius University, Vilnius, Lithuania; 2 Clinic of Internal Diseases, Family Medicine and Oncology, Institute of Clinical Medicine, Faculty of Medicine, Vilnius University, Vilnius, Lithuania

**Keywords:** Acrodysostosis, ACRDYS2, *PDE4D*, Skeletal abnormalities, Intellectual disability, Case report

## Abstract

**Background:**

Acrodysostosis is a rare hereditary disorder described as a primary bone dysplasia with or without hormonal resistance. Pathogenic variants in the *PRKAR1A* and *PDE4D* genes are known genetic causes of this condition. The latter gene variants are more frequently identified in patients with midfacial and nasal hypoplasia and neurological involvement. The aim of our study was to analyse and confirm a genetic cause of acrodysostosis in a male patient.

**Case presentation:**

We report on a 29-year-old Lithuanian man diagnosed with acrodysostosis type 2. The characteristic phenotype includes specific skeletal abnormalities, facial dysostosis, mild intellectual disability and metabolic syndrome. Using patient’s DNA extracted from peripheral blood sample, the novel, likely pathogenic, heterozygous de novo variant NM_001104631.2:c.581G > C was identified in the gene *PDE4D* via Sanger sequencing. This variant causes amino acid change (NP_001098101.1:p.(Arg194Pro)) in the functionally relevant upstream conserved region 1 domain of PDE4D.

**Conclusions:**

This report further expands the knowledge of the consequences of missense variants in *PDE4D* that affect the upstream conserved region 1 regulatory domain and indicates that pathogenic variants of the gene *PDE4D* play an important role in the pathogenesis mechanism of acrodysostosis type 2 without significant hormonal resistance.

## Background

Acrodysostosis (MIM 101800) refers to a group of rare genetic disorders mainly affecting skeletal growth and resulting in primary skeletal dysplasia. Two types of acrodysostosis are known and has been separated by distinct references on OMIM database. Acrodysostosis type 1 with or without hormone resistance (ACRDYS1, MIM 101800) is caused by pathogenic variants in chromosome 17 (locus 17q24.2) of the *PRKAR1A* gene. Acrodysostosis type 2 with or without hormone resistance (ACRDYS2, MIM 614613) is associated with the *PDE4D* gene, which is located in the 5q11.2-q12.1 chromosomal region. According to the new classification proposed by Thiele et al. (2016), ACRDYS1 belongs to the group of inactivating parathyroid hormone (PTH) / PTH-related peptide (PTHrP) signalling disorders 4 (iPPSD4), while ACRDYS2 belongs to iPPSD5 [1]. Acrodysostosis have several overlapping clinical features with another disorder called hypertension and brachydactyly syndrome (HTNB; MIM 112410) classified as iPPSD6. However, this syndrome is caused by heterozygous mutations in *PDE3A* gene which is functionally similar to *PDE4D* [1]. Acrodysostosis is inherited in an autosomal dominant manner. In the literature, most of the patients who have been reported on have de novo pathogenic variants. Characteristic clinical features include skeletal abnormalities, resistance to multiple hormones, and neurological involvement. Skeletal dysplasia is specific and includes disproportional short stature with short extremities and brachydactyly, multiple cone-shaped epiphyses, scoliosis or kyphosis with spinal stenosis, and advanced bone maturation. The facial phenotype is also distinctive and characterized by midfacial and nasal hypoplasia, hypertelorism, epicanthal folds, and low set ears. Patients often show some degree of hormone resistance, including resistance to parathyroid and thyroid stimulating hormones, as well as signs of developmental delay and moderate or mild intellectual disability [2, 3]. Although the two types of acrodysostosis are distinguished primarily on a molecular basis, several clinical differences can also be observed. Patients with pathogenic variants in the *PRKAR1A* gene more frequently have resistance to multiple hormones, while facial dysostosis and neurological involvement are more common in patients with *PDE4D* pathogenic variants [2, 4].

We report on a patient, who has clinical features of acrodysostosis type 2 and a molecularly confirmed novel pathogenic variant in the *PDE4D* gene.

## Case presentation

**Fig. 1:**
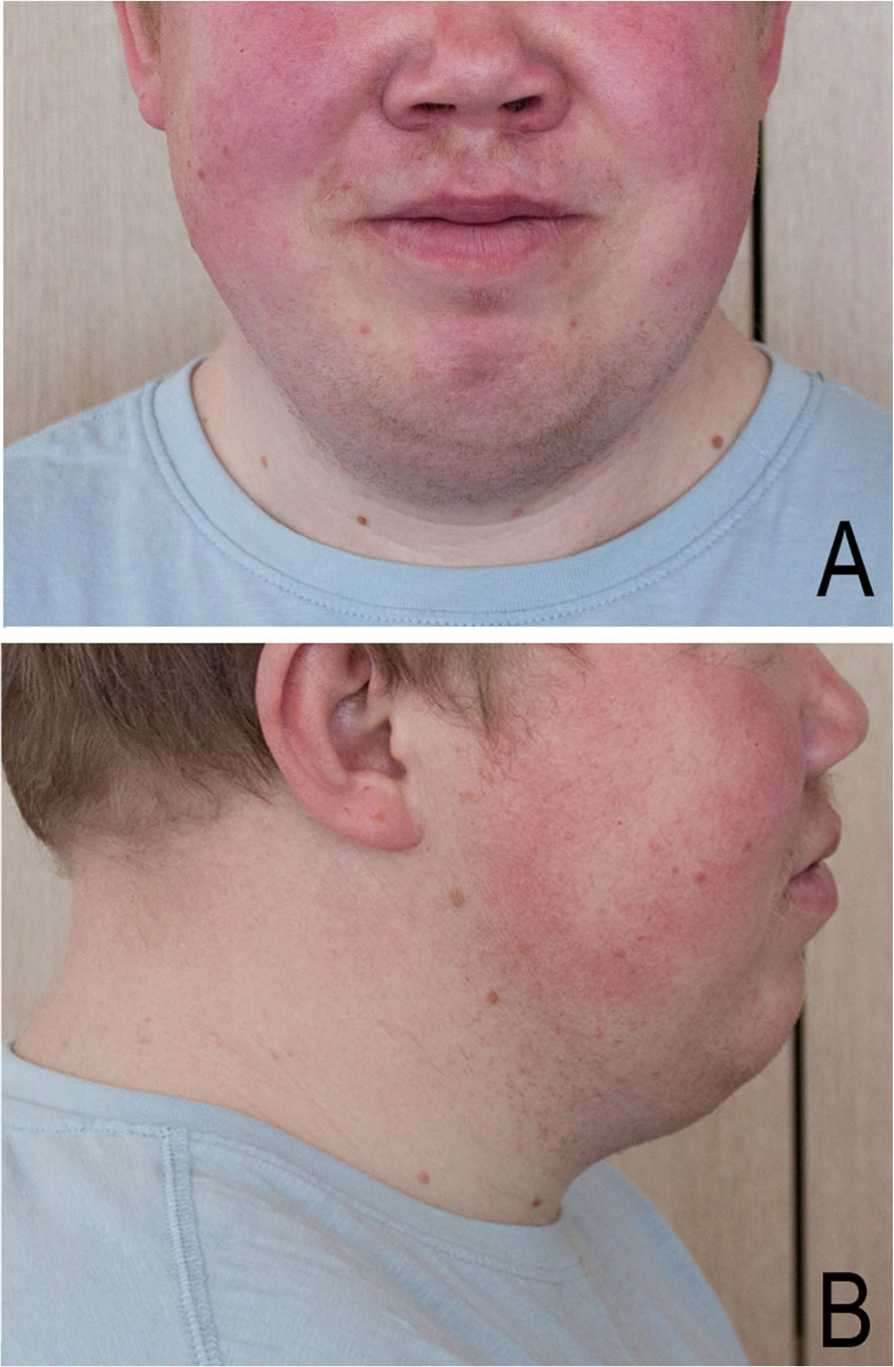
The photographs of the patient at 29 years of age. Note the minor facial anomalies (**a**-**b**), including flat face, depressed nasal bridge, short nose, and long philtrum

**Fig. 2:**
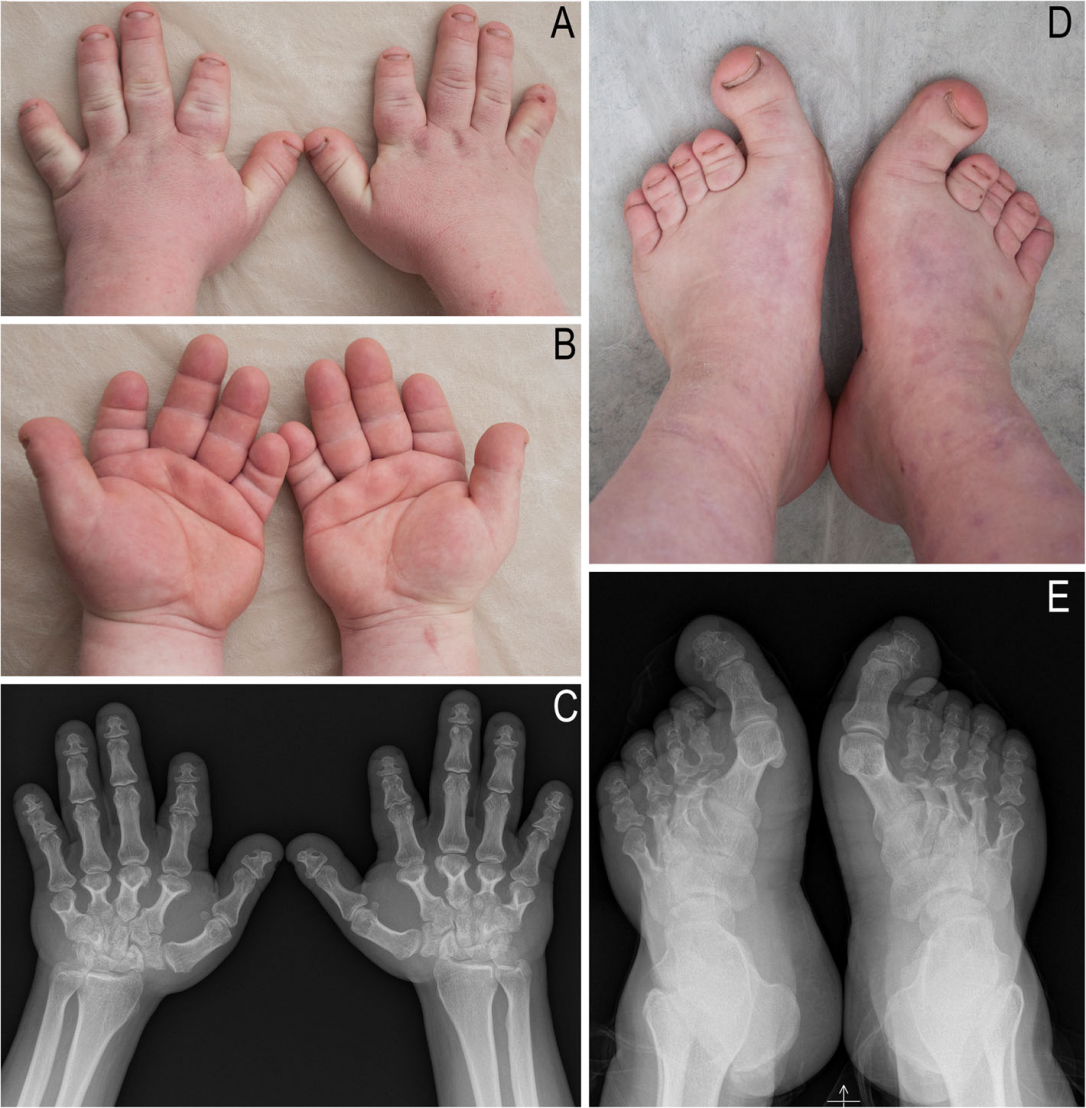
The photographs and X-ray pictures of hands and feet of the patient. The photographs of hands (**a**-**b**) and feet (**d**) show brachydactyly of both hands, a single palmar crease, short rectangular nails, brachydactyly of toes II–V of both feet, short rectangular toenails, and relative hyperplasia of the halluces. X-ray pictures (**c**, **e**) show the short broad metacarpals and phalanges

The 29-year-old male patient is the first child of healthy unrelated Lithuanian parents. The proband was born at full term. Psychomotor development of the proband was delayed. He attended a regular school until the age of 16 years and was further educated in a professional school. An IQ evaluation at the age of 20 showed a score of 69. At his latest evaluation at the age of 29 years, his height was 164 cm, his weight was 85 kg, his BMI was 31 kg/m^2^, and the circumference of his head was 60 cm (90 centile). Other anthropometric measurements were thus: total upper limb length of 66 cm, upper arm length of 35 cm, forearm length of 25 cm, hand length of 15 cm, palm length of 8.5 cm, middle finger length of 6.5 cm, total lower limb length of 87 cm on the left and 86 cm on the right, thigh length of 42 cm on both sides, calf length of 45 cm on the left and 44 cm on the right, and feet length of 23 cm. The patient has minor facial anomalies (Fig. 1), including flat face, sloping forehead with prominent supraorbital ridges, sparse and broad brows, depressed nasal ridge, short nose, long philtrum, and malar hypoplasia. Additionally, dental malocclusion, parodontosis, red and sparse scalp hair, male pattern baldness, and short neck are apparent. The patient is overweight and has limited elbow extension, brachydactyly of both hands, short rectangular nails, a single palmar crease, *valgus* deformation of the knees, brachydactyly of toes II–V of both feet, short rectangular toenails, and relative hyperplasia of the halluces (Fig. 2). The patient complained of premature tiredness, excessive perspiration, and poor fine motor function. He has primary arterial hypertension, and a cardiac ultrasound showed a mitral valve prolapse. An ophthalmological examination revealed hypermetropia. Hormonal screening was remarkable for elevated levels of serum PTH (13.9 pmol/l (*n* = 1.6–7.3 pmol/l)) and somatotropin (12.2 mIU/l (*n* < 9.0 mIU/l)). Elevated PTH levels were probably secondary to vit. D deficiency. TSH, T3, T4, FSH, LH, testosterone, cortisole and ACTH were within normal limits. 25-OH vitamin D was 51.4 nmol/l (*n* = 75–100 nmol/l), serum calcium was 2.58 mmol/l (*n* = 2.10–2.55 mmol/l), serum phosphorus 0.82 mmol/l (*n* = 0.74–1.52 mmol/l), serum glucose was 6.09 mmol/l (*n* = 4.2–6.1 mmol/l). The glucose tolerance test was normal. The ultrasound of the thyroid gland and MRI scan of the hypophyseal gland showed no abnormalities. The X-ray of the hands showed very short and broad tubular bones. Metacarpals II–V are proximally pointed. Coning of phalanges are present in the middle and proximal phalanges of the second to fifth fingers. There is relative sparing of the thumb. The metatarsal bones and phalanges are markedly shortened and there is disproportionate plumpness of the tubular bones of the hallux (Fig. 2). Considering all clinical features and physical examination results, the diagnosis of acrodysostis type 2 was assigned for our proband.

To identify DNA sequence variants in the gene *PDE4D* and analyze segregation, Sanger sequencing was conducted for the proband and his parents. Phenol-chloroform-isoamyl alcohol extraction method was used to isolate genomic DNA from peripheral blood of all three patients. The *PDE4D* exons 2, 3, 6, 7, 13, 15 were amplified by PCR with specific primers (designed using the Primer Blast software, NCBI, USA) and sequenced in the DNA sample of the proband. *PDE4D* exon 2 was amplified and sequenced in the parents’ DNA samples to investigate inheritance pattern of the identified variant. Target PCR products from all three individuals were sequenced with BigDye® Terminator v3.1 Cycle Sequencing Kit (Applied Biosystems, USA) and the capillary electrophoresis was carried out with ABI3130xl Genetic Analyser (Applied Biosystems, USA). Sanger sequencing results were analysed with Sequence Analysis v5.1 software (Applied Biosystems, USA). The reference sequence of the *PDE4D* gene (NCBI: NM_001104631.2) was used for aligning the obtained sequences. Primers and PCR conditions are available from the authors upon request. The pathogenicity of identified DNA sequence variant was evaluated by using several bioinformatics tools: SIFT (https://sift.bii.a-star.edu.sg/), PolyPhen-2 (http://genetics.bwh.harvard.edu/pph2/), MutationTaster2 (http://www.mutationtaster.org/), PROVEAN (http://provean.jcvi.org/about.php). *PDE4D* nucleotide sequence conservation analysis was performed using multiple sequence alignment tool Clustal Omega (EMBL-EBI, https://www.ebi.ac.uk/Tools/msa/clustalo/).

Heterozygous missense variant NM_001104631.2:c.581G > C (NP_001098101.1:p.(Arg194Pro)) located in exon 2 of the gene *PDE4D* was detected in the proband’s DNA sample by Sanger sequencing. Family segregation analysis of the identified variant revealed it’s de novo occurrence. Different in silico tools predicted this variant to be likely pathogenic: SIFT damaging (0,00), PolyPhen-2 (0,99), MutationTaster2 disease causing (0,99), PROVEAN deleterious (− 5,60). The pathogenicity of detected variant was classified according to the guidelines proposed by American College of Medical Genetics and Genomics [5].

## Discussion and conclusions

Pathogenic variants in the *PDE4D* gene were first identified as the cause of acrodysostosis in 2012 [2, 3]. Since then there have been investigations about a possible genotype-phenotype correlation between the two types of acrodysostosis. A few studies showed that ACRDYS1 patients had hormone resistance and short stature, but normal intellect and no facial dysostosis, while the ACRDYS2 group exhibited characteristic facial features, intellectual disability, and no hormone resistance [2, 4]. The distinction between the two types of acrodysostosis was not so obvious in other studies, which suggested that mild short stature with brachydactyly, facial dysostosis, spinal stenosis, a variable degree of developmental disability, and variable endocrine abnormalities may be present in both ACRDYS1 and ACRDYS2 patients [3, 6, 7]. The main clinical features of our patient are mild intellectual disability, facial dysostosis, and specific skeletal abnormalities. Additionally, the presence of increased blood pressure, a documented increased fasting glucose level and abdominal obesity indicated metabolic syndrome. Considering the young age of the patient, we cannot exclude that metabolic syndrome and hypertension are part of this syndrome. Identification of the pathogenic variant in the *PDE4D* gene confirmed the diagnosis of ACRDYS2.

Both *PRKAR1A* and *PDE4D* genes encode proteins essential in the cAMP signaling pathway. PRKAR1A is a crucial part of type I protein kinase A (PKA), the main mediator of cAMP signaling in mammals, while PDE4D is a cAMP specific cyclic nucleotide phosphodiesterase (PDE) that maintains the cellular concentrations of cyclic nucleotides. Cyclic nucleotides are essential messengers that regulate cellular responses to extracellular signals such as hormones or neurotransmitters. Phosphodiesterases (PDEs) contribute greatly to the cAMP signaling pathway and play a role in desensitisation, feedback regulation, and signal compartmentalisation of cells [8]. While there are twelve families of PDEs identified [9], isoforms of the PDE4 family are accountable for most cAMP hydrolysing activity.

The structure of PDE4 consists of a highly conserved catalytic domain in between other domains with regulatory functions. Two of the latter, called upstream conserved regions 1 and 2 (UCR1, UCR2), have been suggested to have major regulatory impact on the PDE4 catalytic unit [10]. UCR1 and linker region 2, which is located between UCR2 and catalytic domain, were detected as the major regions undergoing the process of phosphorylation. This post-translational modification plays a role in regulating the enzymatic activity of PDE4D [11]. Depending on the presence of upstream conserved regions, PDE4 isoforms are divided into categories: long, short, and super-short [9]. Long isoforms have both UCR1 and UCR2, while short isoforms lack UCR1, as do super-short isoforms, which also have truncated UCR2 [12]. When these regulatory domains are defective due to a point mutation of the *PDE4D* gene, the catalytic domain becomes more active and catalysis of cAMP increases abnormally, which leads to the acrodysostosis phenotype. This dominant negative effect of *PDE4D* coding changes has been confirmed in functional studies [6, 13]. On the other hand, haploinsufficiency renders PDE4D completely dysfunctional and results in a mirror phenotype to that of acrodysostosis characterised by long limbs, fingers and toes, specific facial features, and low BMI [6].

**Fig. 3:**
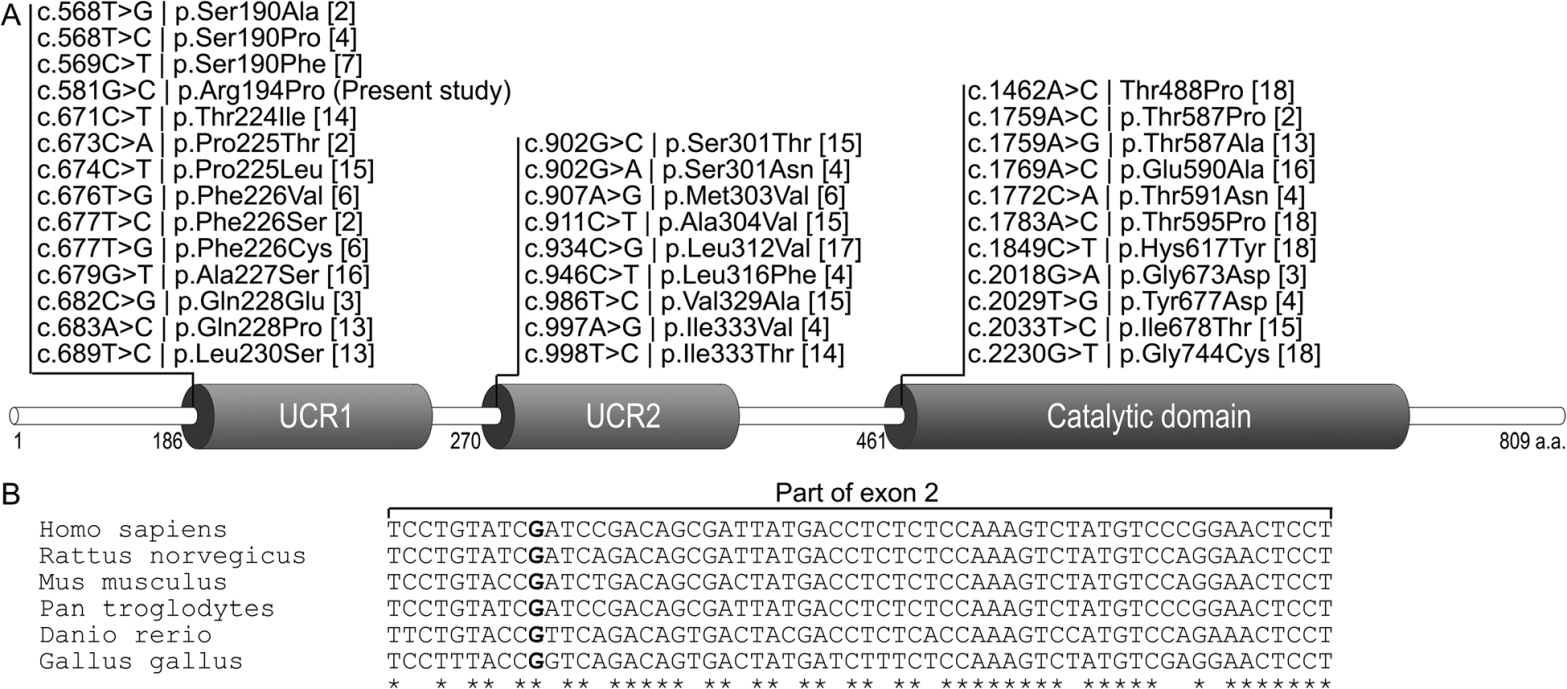
Pathogenic variants of the *PDE4D* gene previously reported in the reviewed literature (**a**). All DNA variants are depicted according to their position in the *PDE4D* gene transcript (NM_001104631.2) and in the PDE4D protein (NP_001098101.1) domains. The novel missense variant of the *PDE4D* gene detected in the present study is also shown. Nucleotide sequence conservation analysis (**b**) showed that altered G nucleotide position is conserved in several animal species (ClustalOmega tool at EMBLEBI website was used)

A novel NM_001104631.2:c.581G > C, NP_001098101.1:p.(Arg194Pro) missense variant detected in our patient affects the UCR1 regulatory domain of the PDE4D protein. The UCR1 functional domain of the protein is the most affected site, followed by catalytic and UCR2 domains, in previously reported, affected patients (Fig. 3a) [2–4, 6, 7, 13–18]. The missense c.581G > C variant was not observed in approximately 6300 individuals of European and African American ancestry in the NHLBI Exome Sequencing Project, indicating it is not a common benign variant in these populations. It leads to nonsynonymous substitution of positively charged arginine to nonpolar proline. This substitution occurs at a position that is conserved across species (Fig. 3b), and in silico analysis predicts this variant is probably damaging to protein function. Healthy parents have been confirmed to not possess the same variant, which further contributes to the notion of its pathogenicity. Four missense variants located more proximally in the UCR1 have been previously reported, supporting the functional importance of this region of the protein [1]. Other *PDE4D* gene variants that have been described to date are presented in Fig. 3a.

In summary, this study adds to the literature a novel pathogenic *PDE4D* missense variant that results in clinical features specific to acrodysostosis type 2 without significant hormone resistance. By reviewing the literature and combining the clinical and molecular findings of our patient with previously published data, we further expand the knowledge of the consequences of missense variants in the *PDE4D* gene that affect the UCR1 regulatory domain.

## Data Availability

The dataset of the exon 2 of the gene *PDE4D* chromatograms generated and analysed during the current study is available in Zenodo repository (10.5281/zenodo.4644688). Any additional information is available from the authors upon request.

## Abbreviations

ACRDYS1: Acrodysostosis type 1
ACRDYS2: Acrodysostosis type 2
ACTH: Adrenocorticotropic hormone
BMI: Body mass index
cAMP: Cyclic adenosine monophosphate
FSH: Follicle-stimulating hormone
HTNB: Hypertension and brachydactyly syndrome
iPPSD4: Inactivating parathyroid hormone / PTH-related peptide signalling disorders 4
iPPSD5: Inactivating parathyroid hormone / PTH-related peptide signalling disorders 5
iPPSD6: Inactivating parathyroid hormone / PTH-related peptide signalling disorders 6
LH: Luteinizing hormone
MRI: Magnetic resonance imaging
NHLBI: National Heart, Lung and Blood Institute
PCR: Polymerase chain reaction
PDE: Acronym for proteins phosphodiesterases
PDE4: A proteins family of phosphodiesterases 4
*PDE3A*: Gene symbol for gene that encodes a phosphodiesterase 3A
*PDE4D*: Gene symbol for gene that encodes a protein phosphodiesterase 4D
PKA: Protein kinase A
*PRKAR1A*: Gene symbol for gene that encodes protein kinase cAMP-dependent type I regulatory subunit alpha
PTH: Parathyroid hormone
PTHrP: PTH-related peptide
T3: Triiodothyronine
T4: Thyroxine
TSH: Thyroid-stimulating hormone
UCR1: Upstream conserved region 1, a regulatory domain of protein PDE4D
UCR2: Upstream conserved region 2, a regulatory domain of protein PDE4D
